# Healthcare Professionals’ Compliance with the Standard Management Guidelines towards the Use of Biological Disease-Modifying Anti-Rheumatic Drugs in Rheumatoid Arthritis Patients

**DOI:** 10.3390/ijerph19084699

**Published:** 2022-04-13

**Authors:** Sadia Shakeel, Wajiha Iffat, Ambreen Qamar, Hina Rehman, Faiza Ghuman, Fareeha Butt, Anees ur Rehman, Melinda Madléna, Edit Paulik, Márió Gajdács, Shazia Jamshed

**Affiliations:** 1 Department of Pharmacy Practice, Faculty of Pharmaceutical Sciences, Dow College of Pharmacy, Dow University of Health Sciences, Karachi 74200, Pakistan; 2 Department of Pharmaceutics, Faculty of Pharmaceutical Sciences, Dow College of Pharmacy, Dow University of Health Sciences, Karachi 74200, Pakistan; wajiha.iffat@duhs.edu.pk; 3 Department of Physiology, Dr. Ishrat Ul Ebad Khan Institute of Oral Health Sciences (DIKIOHS), Dow University of Health Sciences, Karachi 74200, Pakistan; ambreen.qamar@duhs.edu.pk; 4 Department of Pharmacy Practice, Institute of Pharmaceutical Sciences, Jinnah Sind Medical University, Karachi 75510, Pakistan; hina.rehman@jsmu.edu.pk; 5 Department of Medicine, Dow University Hospital, Dow University of Health Sciences, Karachi 74200, Pakistan; faizulmaram@hotmail.com; 6 Department of Physiology, Dow Medical College, Dow University of Health Sciences, Karachi 74200, Pakistan; fareeha.butt@duhs.edu.pk; 7 Department of Pharmacy Practice, Faculty of Pharmacy, Bahauddin Zakariya University, Multan 60800, Pakistan; aneesurrehmanr90@gmail.com; 8 Department of Oral Biology and Experimental Dental Research, Faculty of Dentistry, University of Szeged, 6720 Szeged, Hungary; madlena.melinda@stoma.szote.u-szeged.hu; 9 Department of Public Health, Faculty of Medicine, University of Szeged, 6720 Szeged, Hungary; paulik.edit@med.u-szeged.hu; 10 Department of Clinical Pharmacy and Practice, Faculty of Pharmacy, Universiti Sultan Zainal Abidin, UniSZA, Kuala Terengganu 21300, Malaysia; shaziajamshed@unisza.edu.my

**Keywords:** rheumatoid arthritis, disease-modifying anti-rheumatic medications, healthcare professionals, EULAR guidelines, Pakistan

## Abstract

Treatment of rheumatoid arthritis (RA) is complicated, with numerous aspects influencing decision-making, including disease severity, comorbidities, and patient preferences. The present study aimed to evaluate healthcare professionals’ (HCPs) knowledge of biological disease-modifying anti-rheumatic drugs (bDMARDs) and their compliance with the standard management guidelines for assuring optimal RA therapy. The cross-sectional, survey-based study was performed in various healthcare and academic settings in Karachi, Pakistan to probe HCPs’ knowledge of bDMARDs and their compliance with the European League against Rheumatism (EULAR) recommendations for the management of RA patients. Overall, *n* = 413 questionnaires were included in our study (response rate: 82.6%). The physicians were further well-informed about the indications (*n* = 276, 91.3%, *p* = 0.001) and monitoring requirements (*n* = 258, 85.4%, *p* = 0.004). The pharmacists were more knowledgeable about the drug targets (*n* = 96, 86.4%, *p* = 0.029) and their mechanisms of action (*n* = 80, 72.0%, *p* = 0.013). Male respondents as compared with females (41.3% vs. 35.6%, *p* = 0.04), and physicians as compared with pharmacists (40.7% vs. 37.8%, *p* = 0.012), were more confident in using bDMARDs than conventional treatment in RA patients. Our findings show that the respondents were familiar with the attributes of bDMARDs and the standard management guidelines for RA care. Our results may be relevant in creating new methods, guidelines, and treatments to enhance RA treatment adherence, satisfaction, and health outcomes.

## 1. Introduction

Persistent musculoskeletal pain is a global health issue that has a detrimental impact on people’s well-being. Osteoarthritis (OA), rheumatoid arthritis (RA), and spine-related neck and back problems are the most frequent musculoskeletal ailments. Musculoskeletal problems are the leading cause of disability in working-age individuals [1]. RA is a common chronic inflammatory joint disorder with a heterogeneous disease presentation, accompanied by various comorbidities, such as higher cardiovascular risk, diabetes, irritable bowel disease (IBD), blood clots, and sleep apnea, which may lead to increased morbidity and mortality [2]. As a result, optimal disease management is critical for preventing disease progression in RA and for improving long-term patient outcomes. There is no unambiguous biomarker that allows for the selection of the medicines best suited for each individual patient [3]. The treatment of RA necessitates the use of various medications with distinct mechanisms of action to accomplish remission or—at the very least—minimal disease activity, as indicated by the recommendations of the European League against Rheumatism (EULAR) [4]. In the recent two decades, there has been a revolution in therapeutic armamentarium for RA, which includes a treatment-to-target approach and novel biological and non-biological anti-rheumatic drugs [5]. All professional rheumatology organizations have agreed on the standard of therapy for RA, which involves the early administration of disease-modifying anti-rheumatic drugs (DMARDs) [6]. Treatment objectives must include attaining remission or minimal disease activity, along with a decrease in the radiographic progress of the illness. The onset of treatment in these patients needs to occur as soon as possible to prevent the loss of joint function, structural joint deterioration, and maintaining quality of life (QoL) in the affected individuals [7].

DMARDs are immunosuppressive and immunomodulatory medicines that may either be conventional or biologic in nature. Methotrexate, hydroxychloroquine, leflunomide, and sulfasalazine are examples of commonly used conventional DMARDs (csDMARDs) [8]. Biologic DMARDs (bDMARDs) were introduced into clinical practice in the early 1990s, and are often used when standard DMARD therapy has failed [7]. Treatment for RA may be either combination therapy or monotherapy; however, several randomized controlled trials have indicated that combining bDMARDs with csDMARDs leads to significantly better outcomes in patients than using either of these drugs alone [9]. As biologics target particular mediators of RA inflammation, they may be more effective than traditional DMARDs in managing the progression of RA. These newer bDMARDs, unlike csDMARDs, are produced utilizing biotechnology. They have been genetically modified to function like natural proteins in the immune system. Some of these medications act by disrupting specific chemical signals involved in inflammation, while others work directly on T-cells or B-cells to disrupt the inflammatory process. Many biologics function by interfering with the action of tumor necrosis factor (TNF), a major protein in the immune system. They slow the development of RA by altering the body’s biochemical response to different cytokines—most notably TNF-α, which is the main cytokine responsible for systemic inflammation and is elevated in patients with RA [10]. As a result, bDMARDs may be classified as either anti-TNF biologics or non-TNF biologics.

The bDMARDs are specialized and target a particular pathway of the immune system [11]. Their overarching roles include blocking the secondary signals needed for T-cell activation, interfering with cytokine synthesis or function, and reducing or inhibiting substances that activate B-cells [12]. Although the combination of a bDMARD and a csDMARD, as well as the usage of multiple csDMARDs, is considered safe, the use of a combination of different bDMARDs is not recommended owing to an augmented risk of immunosuppression, which may lead to potentially fatal opportunistic infections. The concept of indicator opportunistic infection after biological therapy has recently been raised in response to consensus recommendations of the existence, or specific presentation, of a pathogen that implies a higher chance of a modification in immunity in a host receiving bDMARDs [13]. The most common adverse event associated with the use of all bDMARDs is the higher risk of viral, bacterial, or fungal infections; in addition to this, chronic tuberculosis (TB), hepatitis B (HBV) and C (HCV), and herpes zoster virus (HHV3) may be reactivated. Patients must be tested for HBV and HCV infections, while screening for TB is strongly advised before the initiation of a DMARD treatment [14]. On rare occasions, specific agents have been linked to bone marrow suppression and hepatotoxicity. Anti-TNF medicines may aggravate drug-induced lupus, congestive heart failure, and demyelinating central nervous system (CNS) disorders [15].

The management of RA is complicated, with numerous aspects influencing decision-making for clinicians, including comorbidities, disease severity and activity, and patient preferences [16]. RA management guidelines suggest that DMARDs should preferably be prescribed by a qualified expert, such as a rheumatologist, owing to the considerable complexity required in the effective use of these drugs and potential adverse effects [17]. The prescribing physician must be competent in their knowledge of the indications and possible side effects of these medicines, and should consult with the pharmacist about dosing and potential drug–drug interactions. Nursing staff may likewise collaborate with the pharmacists and physicians in patient counseling and monitoring, as all patients receiving DMARDs must be continuously monitored for efficacy and adverse events [16,18]. Hence, it is important to investigate the characteristics associated with healthcare professionals’ (HCPs) approach to the management of RA. The purpose of the present study was to explore HCPs’ knowledge of bDMARDs and their compliance with the standard management guidelines for assuring optimal RA therapy for their patients.

## 2. Materials and Methods

### 2.1. Study Design and Setting

The cross-sectional, survey-based study was performed between June and August 2021 (12 weeks). The responses were collected from different public and private sector healthcare and academic settings in Karachi, Pakistan. The respondents were approached through email or personal contacts and invited to complete the survey form. The approach of convenience sampling was used, and the respondents were selected based on their ease of availability and proximity.

### 2.2. Study Population and Sampling

The respondents were either physicians or pharmacists and considered eligible only if the following conditions were met:

- Those who were registered with the appropriate professional body for accreditation;
- Those who provided a written consent form for their voluntary participation in the study.

The Raosoft sample size calculator was used to compute the sample size needed for this study, based on the Formula (1) below:

(1) $$n=N\frac{x}{\left(N-1\right)E2+x}$$

where the population is *N* = 20,000 (as we do not know exactly the total number of physicians and pharmacists in Karachi), *x* is the confidence interval of 95%, *E* is the margin of error set at 5%, and the expected response rate is set at 50% [19]; based on this, the expected response rate was found to be *n* = 377.

### 2.3. Study Instrument

To design the questionnaire utilized in this study, a comprehensive literature analysis of previous studies was undertaken in the PubMed/MEDLINE database to generate potential questions. After an extensive literature review of the previous studies published on the topic and the recommended guidelines for the management of RA patients, the study tool consisting of 30 items was developed in English language [3,17,20,21]. The face validity of the developed questionnaire was assessed by three physicians and one pharmacist from the Dow University of Health Sciences. The questionnaire was than directed to *n* = 30 respondents (including 23 general physicians and 7 pharmacists) to evaluate the content validity and, thereby, to ascertain the consistency and acceptability of the questionnaire items. Slight modification was desired after the pilot testing. The internal consistency of the questionnaire was determined by calculating the Cronbach’s alpha value, which was determined to be within acceptable limits (α  =  0.7). The data obtained from the respondents included in the pilot study were excluded from the final analysis. It was then distributed through email or personal contacts among the potential respondents for data collection.

Along with the six questions associated with the respondents’ baseline information, the survey form consisted of three sections: The first section comprised of 10 close-ended questions with “true”, “false”, and “do not know” options to examine respondents’ understanding of bDMARDs. The second section included twelve questions corresponding to the EULAR recommendations for the management of RA. The items were scored on a five-point Likert scale, ranging from strongly agree = 5 to strongly disagree = 1, with the aim of assessing respondents’ attitudes and perceptions towards the initiation of therapy with DMARDs, treatment targets, their practices of shared decision-making, prospects of substituting bDMARDs, and the inclination of using bDMARDs in clinical practice. The third section included questions about the respondents’ concerns towards the safety and efficacy of bDMARDs, their information sources to decide on the use of biosimilar products, their perceived significant factors, and barriers of using bDMARDs in their clinical practice.

### 2.4. Data Collection

The survey-based data were gathered from HCPs working in various healthcare and academic settings in Karachi. Respondents were provided with the questionnaire after being informed about the study’s purpose, benefits, and risks. The questionnaires were filled out anonymously by the respondents. The questionnaires, together with the consent form, were collected later, as per the convenience of the respondents. Respondents were neither compensated nor offered any gifts in exchange for their participation in the survey. All questionnaires were carefully reviewed, and those with a completion rate of more than 90% were included in the analysis. The responses of the HCPs were kept anonymous and voluntary.

### 2.5. Data Analysis

Descriptive statistics were used to evaluate the baseline characteristics of the respondents. Continuous data were expressed as means and standard deviations (SDs), while categorical variables were expressed as percentages. To determine the type of data distribution, the Kolmogorov–Smirnov test was used. To analyze statistical differences among groups, inferential statistics (Mann–Whitney U test and Kruskal Wallis tests) were employed, considering a *p*-values of less than 0.05 as significant. SPSS for Windows version 24.0.0 (SPSS, IBM Inc., Chicago, IL, USA) was used for data analysis.

## 3. Results

### 3.1. Demographic Characteristics

Among the respondents approached for the study, *n* = 23 physicians and *n* = 9 pharmacists did not show a willingness to participate. Seventeen survey forms were omitted from the study, as the consent forms had not been duly filled out. Lastly, *n* = 413 completed surveys were incorporated into the investigation, with a response rate of 82.6%. The respondents’ mean age was 31.8 ± 12.8 years. Among them, 33.4% (*n* = 138) were males and 66.5% (*n* = 275) were females. The physicians made up 73.1% (*n* = 302), with a majority of them working in internal medicine, whereas 26.8% (*n* = 111) were pharmacists. The detailed demographic information of the respondents is demonstrated in Table 1.

### 3.2. Assessment of Knowledge of Respondents Regarding bDMARDs

Table 2 outlines the respondents’ knowledge regarding bDMARDs. In total, 36.3% (*n* = 150) and 32.4% (*n* = 134) of the respondents considered themselves to be extremely and moderately familiar with the recent developments in bDMARDs, respectively. Their level of familiarity was significantly associated with their gender (*p* = 0.03), professional specialty (*p* = 0.005), and working organization (*p* = 0.002), while a numerical tendency was observed in association with experience (*p* = 0.057). The physicians were more well-informed regarding the indications (*n* = 276, 91.3%; *p* = 0.001) and monitoring requirements (*n* = 258, 85.4%; *p* = 0.004). The pharmacists were more informed regarding the targets of the drugs (*n* = 96, 86.4%; *p* = 0.029) and their mechanisms of action (*n* = 80, 72.0%; *p* = 0.013) as compared with physicians. Experienced respondents were more informed about the examples of disease-modifying anti-rheumatic drugs (*p* = 0.001), drug targets (*p* = 0.004), monitoring requirements (*p* = 0.003), and the general precautions associated with them (*p* = 0.001). The majority of physicians (74.8%, *n* = 226) and pharmacists (71.1%, *n* = 79) were aware that, before using bDMARDs, the functional capacity and damage to the joints, disease progression, pregnancy status, other comorbidities, extra-articular manifestations, and immunization history should be evaluated in their patients. Similarly, around two-thirds of the respondents, i.e., 70.8% (*n* = 214) of physicians and 75.6% (*n* = 84) of pharmacists, were aware that all patients should be screened for active or latent TB and HBV infections before initiating therapy with bDMARDs.

### 3.3. Respondents’ Compliance with the RA Standard Management Guidelines

On inquiring about the respondents’ compliance with the RA standard management guidelines, 66.0% (*n* = 273) of the respondents agreed that DMARD therapy should be initiated immediately upon the RA diagnosis being confirmed. (Table 3) The respondents working in public sector organizations were more inclined towards the immediate use of DMARDs as compared with the respondents working in private healthcare organizations (70.5% vs. 56.6%, *p* = 0.018). The majority of them (71.3%, *n* = 205) stated that, if therapy with an initial bDMARD is unsuccessful, substituting another bDMARD could be needed. The pharmacists were more willing to substitute with another bDMARD as compared with physicians (80.1% vs. 66.5%, *p* = 0.001). Around 80% of the respondents agreed that, in the event of not accomplishing the treatment objective with the first csDMARD, other csDMARDs should be considered or a bDMARD should be added. More than 75% of the respondents agreed that, if the use of methotrexate is contraindicated in a patient, leflunomide or sulfasalazine should be included in the initial treatment strategy.

The respondents having clinical experience of more than ten years were more likely to state that bDAMRDs should only be prescribed by experienced rheumatologists (*p* = 0.009) and that patients should be educated about their therapy with bDMARDs (*p* = 0.028). Slightly more than half of the respondents (58.1%; *n* = 240) stated that the patient should engage in collaborative decision-making when opting to employ bDMARDs in their therapy. Male respondents as compared with females (41.3% vs. 35.6%, *p* = 0.04), and physicians as compared with pharmacists (40.7% vs. 37.8%, *p* = 0.012), were more confident in using bDMARDs than using conventional treatment in RA patients. More than 70% of the respondents showed their likelihood of using bDMARDs in their practice setting once approved by the U.S. Food and Drug Administration (FDA).

### 3.4. Respondents’ Perceived Importance and Barriers of Using bDMARDs

**Figure 1:**
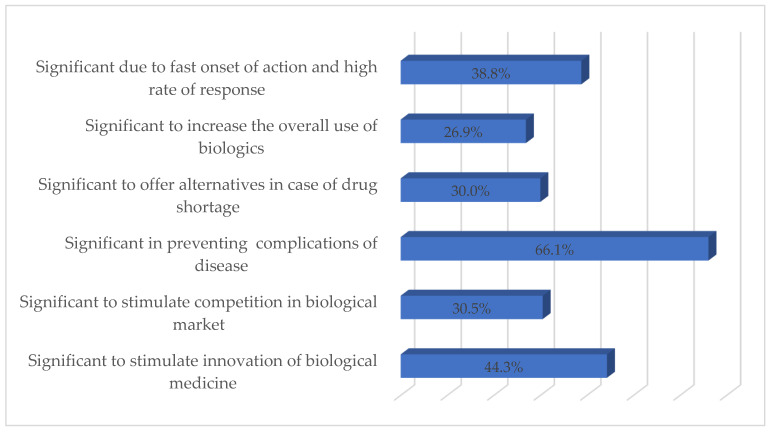
Respondents’ perceived importance of bDMARDs (%).

Figure 1 shows the respondents’ perceived importance of bDMARDs in the treatment of RA. The majority of respondents (66.1%; *n* = 273) considered bDMARDs effective in controlling symptoms and preventing complications of RA, whereas 44.3% (*n* = 183) of respondents deemed bDMARDs important to stimulate innovation in biological medicine. Approximately 30% of the respondents consider bDMARDs important to encourage competition in the biological market and as an effective alternative in the event of a drug shortage.

**Figure 2:**
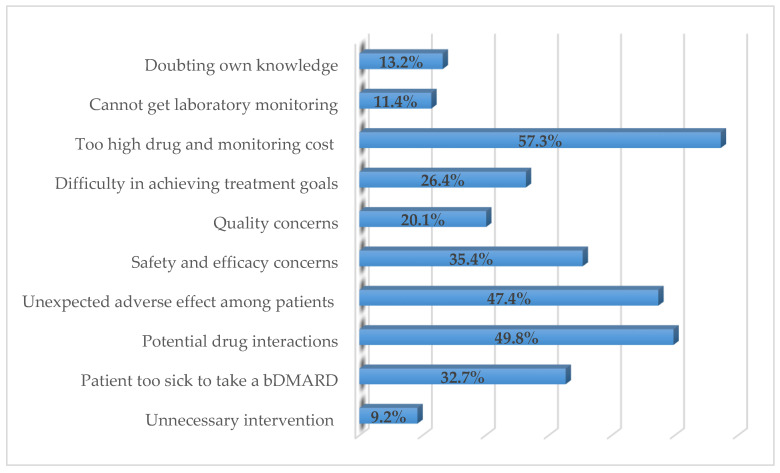
Respondents’ perceived barriers to prescribing/dispensing bDMARDs in RA patients (%).

**Figure 3:**
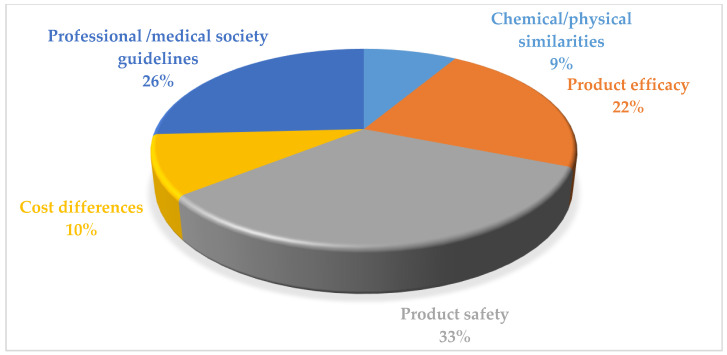
Respondents’ perceived important sources to decide the use of biosimilar products (%).

Figure 2 illustrates respondents’ perceived barriers to prescribing and dispensing bDMARDs in RA patients. The main barriers reported by the respondents were too high drug and monitoring costs (57.3%; *n* = 237), potential drug–drug interactions (49.8%; *n* = 206), and unexpected adverse effects among patients (47.4%; *n* = 196). Product safety (33.1%; *n* = 137), professional/medical society guidelines (26.3%; *n* = 109), and the products’ efficacy (22%; *n* = 91) were the respondents’ perceived important sources to decide on the use of biosimilar products (Figure 3).

## 4. Discussion

The current study provided a glimpse into HCPs’ knowledge of bDMARDs and their compliance with the standard management guidelines for the management of RA using DMARDs. The research conducted so far depicting HCPs’ prescribing patterns and compliance with standard guidelines for RA care has been scarce, and ours is the first questionnaire-based study on the issue among Pakistani HCPs, specifically among physicians and pharmacists. To be proactive in the RA treatment strategy, HCPs must be aware of, understand, and agree with the standard recommendations of the guidelines [1]. The current study found that HCPs involved in the study had an awareness of bDMARDs, with the majority of the respondents knowing the drug targets, mechanism of action, monitoring requirements, and general principles of using bDMARDs. The current study had a response rate of 82.6%, which may be deemed adequate given other findings in the literature [22]. The majority of respondents were aware that, before starting bDMARDs, all patients with inflammatory arthritis should have their disease activity, joint functional ability and damage, extra-articular symptoms, co-morbidities, immunization history, and pregnancy status evaluated. Other studies likewise stated that the patient’s characteristics and the patient’s disease presentation influenced the decision to prescribe biologics to a greater extent [17,23]. Despite the revelation that RA therapies may decrease the immune response to specific vaccines, such as those against hepatitis B, invasive *Streptococcus pneumoniae* (pneumococcus), and influenza, people with RA should continue to get certain immunizations to limit the occurrence of infections [24].

In RA patients taking methotrexate or biologics, the standard recommendations for the use of biologic and non-biologic treatment explicitly recommend immunization against pneumococcus, hepatitis B, and influenza [25]. Vaccination against herpes zoster may be administered to patients taking methotrexate, but should be avoided in patients receiving biological treatment. Furthermore, in patients receiving biologic treatment, all live virus vaccinations should be avoided [24]. The majority of the respondents, i.e., physicians and pharmacists, were aware that, before starting bDMARDs, there is a need to screen all patients for HBV and latent or active TB. More than 70% of the respondents showed likelihood of using bDMARDs in their practice setting, once approved by the U.S. FDA. Another study also reported the respondents’ inclination to use csDMARDs, with 44% of prescribers reporting using bDMARDs [23].

In the present study, more than 65% of the respondents agreed that DMARD therapy should be started as soon as the RA diagnosis is confirmed. One more study inquiring into respondents’ opinions on the best period to start DMARD therapy reported that some HCPs indicated ‘within the first 6 months of diagnosis’, while 35% indicated ‘after a trial of NSAIDs or steroids’, and 5% indicated ‘at least 6 months after diagnosis’ [23]. In over 30 years, the management of RA has been fundamentally transformed as data have been accumulated to support the efficacy of DMARDs in improving outcomes [26]. The goal of RA therapy has since been shifted from symptom management to disease modification. The use of DMARDs at an early stage is currently suggested to slow disease development and prevent long-term impairment. As DMARD initiation is the domain of experts in secondary care, early usage necessitates early referral [27]. The current research showed that the experienced respondents were more inclined to believe that bDAMRDs should only be provided by a rheumatologist, who is experienced in diagnosing and managing rheumatic diseases. A study in Ontario, Canada used a case scenario to investigate physicians’ care of RA, and discovered that most doctors would correctly diagnose RA; nevertheless, referral rates to specialists are uncommon [23].

It would have been difficult to assess the respondents’ adherence to the RA standard care recommendations, as every patient may have a distinct course of illness, which may influence the physicians’ management decisions. A treatment decision might have long-term implications, as well as significant uncertainty and compromises. One of the important competencies required for patients to participate in treatment decisions is the capacity to interpret risk information [28]. The purpose of risk communication in the context of selecting a new drug is to promote informed decisions with increased patient autonomy [29]. Slightly more than half of our respondents considered that the patient should be engaged in collaborative decision-making when choosing to employ bDMARDs for their therapy. Mahlich et al. found that nearly half of the respondents were persuaded on the necessity of patients’ involvement in mutual decision-making [30]. A study described respondents’ opinion that the use of biosimilar products boosts patients’ accessibility to a range of treatment alternatives and stimulates competition in manufacturers, which contributes to lower product pricing [31]. A similar opinion of the respondents was observed in the present study. The respondents’ perceived barriers to prescribing/dispensing bDMARDs in RA patients were as follows: too high drug and monitoring costs, potential drug interactions, and unexpected adverse effects among patients. Garneau et al. likewise reported an uncomfortable attitude of the majority of respondents when initiating a DMARD therapy [23]. The most common reasons reported for their reluctance to use DMARD were ‘toxicities’, ‘infections’, and ‘intravenous treatment’. Contrarily, Kalkan et al. showed that the economic resources and medication cost were all regarded as influencing the prescription choice [32]. Financial reasons, such as budget responsibility, have been cited in an earlier study as having an impact on the adoption of novel treatments [33]. According to a recent systematic analysis, the sole presence of financial considerations may impact prescriptions [34]. The current study stated that product safety, professional/medical society guidelines, and product efficacy were the respondents’ perceived important sources for deciding on the use of biosimilar products. A recent systematic review reported that the available data on medication effects were characterized as having an important influence on prescription choices [35]. The literature has also reported that colleagues and clinical meetings were, in many cases, the primary information sources for physicians [36].

Several limitations may have an impact on the current study’s findings. To begin with, HCPs’ recommendations were employed as the sole measure of adherence in the current study. Furthermore, the nature of the study was cross-sectional. Ideally, HCPs’ compliance should be assessed using a more comprehensive indicator that considers the kind of therapy, attainment of treatment objectives, follow-up, and monitoring.

## 5. Conclusions

The present findings revealed that HCPs were well-versed with the attributes and uses of bDMARDs and the standard management guidelines for RA care. The outcomes may have provided a platform for identifying disparities between current management practices and standard guidelines, which may be valuable in designing future initiatives to standardize and deliver optimal RA patient care.

## Figures and Tables

**Table 1:** Characteristics of the study population.

Baseline Characteristics	Frequency *n* (%)
**Age (Years)**	
Mean ± SD	31.8 ± 12.8
**Gender**	
Male	138 (33.4)
Female	275 (66.5)
**Organization**	
Private	158 (38.3)
Public sector	255 (61.7)
**Professional Specialty**	
Physicians	302 (73.1)
Pharmacists	111 (26.8)
**Practice Area**	
Primary patient care	110 (26.6)
Secondary patient care	28 (6.7)
Tertiary patient care	275 (66.5)
**Experience**	
<5 years	239 (57.8)
5–10 years	68 (16.4)
10–15 years	66 (15.9)
15–20 years	30 (7.2)
20 years and above	10 (2.4)

**Table 2:** Respondents’ knowledge regarding bDMARDs.

Knowledge Item	Correct Response		Gender	Organization	Specialty	Practice Area	Experience
	Physicians (*n* = 302)	Pharmacists (*n* = 111)					
Indications and use	276 (91.3)	99 (89.1)			0.001 *		
Types/examples	244 (80.7)	93 (83.7)					0.001 *
Drug target	171 (56.6)	96 (86.4)	0.005 *		0.029 *		0.004 *
Mechanism of action	160 (52.9)	80 (72.0)			0.013 *		
General principles for use	226 (74.8)	79 (71.1)		0.007 *		0.001 *	
Monitoring requirements	258 (85.4)	82 (73.8)			0.004 *		0.003 *
Contraindications	195 (64.5)	71 (63.9)					
General precautions	214 (70.8)	84 (75.6)	0.002 *				0.001 *
Benefits and risks	189 (62.5)	88 (79.2)		0.001 *			
Adverse effects	177 (58.6)	86 (77.4)					

* The difference in responses was statistically significant (*p* < 0.05).

**Table 3:** Respondents’ compliance with the RA standard management guidelines.

To What Extent Do You Agree or Disagree with the Following Statements?	Strongly Agree *n* (%)	Agree *n* (%)	Neutral *n* (%)	Disagree *n* (%)	Strongly Disagree *n* (%)
DMARD therapy should initiated immediately upon the RA diagnosis being confirmed.	101 (24.4)	172 (41.6)	97 (23.4)	21 (5.0)	22 (5.3)
Patients need access to multiple medications with different modes of action to combat the heterogeneity of RA; they may need multiple consecutive therapies during their life course.	147 (35.5)	168 (40.6)	56 (13.5)	33 (7.9)	9 (2.1)
Monitoring should be regular in active disease and therapy should be adjusted in the case of no improvement.	103 (24.9)	167 (40.4)	125 (30.2)	13 (3.1)	5 (1.2)
Methotrexate should be included in the initial treatment strategy.	56 (13.5)	243 (58.8)	86 (20.8)	25 (6.0)	3 (0.7)
If the use of methotrexate is contraindicated in a patient, leflunomide or sulfasalazine should be included in the initial treatment strategy.	164 (39.7)	152 (36.8)	88 (21.3)	5 (1.2)	4 (0.9)
Short-term glucocorticoids should be considered when initiating or changing csDMARDs.	103 (24.9)	168 (40.6)	116 (28.0)	22 (5.3)	4 (0.9)
If the treatment target is not accomplished with the first csDMARD, other csDMARDs strategies should be considered.	106 (25.6)	227 (54.9)	70 (16.9)	7 (1.6)	3 (0.7)
If the treatment target is not achieved with the first csDMARD strategy, then a bDMARD should be added.	122 (29.5)	201 (48.6)	66 (15.9)	21 (5.0)	3 (0.7)
bDMARDs should be combined with a csDMARD in patients who cannot use csDMARDs as a co-medication.	150 (36.3)	132 (31.9)	120 (29.0)	5 (1.2)	6 (1.4)
If a bDMARD has failed, treatment with another bDMARD should be considered.	104 (25.1)	191 (46.2)	85 (20.5)	19 (4.6)	14 (3.3)
Treatment goals of RA patients should be based on a shared decision between the patient and the rheumatologist.	118 (28.5)	122 (29.5)	129 (31.2)	39 (9.4)	5 (1.2)
Treatment decisions are based on disease activity, safety issues, and other patient factors such as co-morbidities and progression of structural damage.	101 (24.4)	184 (44.5)	81 (19.6)	18 (4.3)	29 (7.0)

## Data Availability

All data generated during the study are presented in this paper.

## References

[1] Conigliaro P., Triggianese P., De Martino E., Fonti G.L., Chimenti M.S., Sunzini F., Viola A., Canofari C., Perricone R. (2019). Challenges in the treatment of rheumatoid arthritis. Autoimmun. Rev..

[2] Chaudhari K., Rizvi S., Syed B.A. (2016). Rheumatoid arthritis: Current and future trends. Nat. Rev. Drug Discov..

[3] Agca R., Heslinga S., Rollefstad S., Heslinga M., McInnes I., Peters M.J.L., Kvien T.K., Dougados M., Radner H., Atzeni F. (2017). EULAR recommendations for cardiovascular disease risk management in patients with rheumatoid arthritis and other forms of inflammatory joint disorders. Ann. Rheum. Dis..

[4] Aletaha D., Smolen J.S. (2018). Diagnosis and management of rheumatoid arthritis: A review. JAMA.

[5] Walsh D.A., McWilliams D.F. (2012). Pain in rheumatoid arthritis. Curr. Pain Headache Rep..

[6] Fautrel B., Den Broeder A.A. (2015). De-intensifying treatment in established rheumatoid arthritis (RA): Why, how, when and in whom can DMARDs be tapered?. Best Pract. Res. Clin. Rheumatol..

[7] Abbasi M., Mousavi M.J., Jamalzehi S., Alimohammadi R., Bezvan M.H., Mohammadi H., Aslani S. (2019). Strategies toward rheumatoid arthritis therapy; the old and the new. J. Cell. Physiol..

[8] Friedman B., Cronstein B. (2019). Methotrexate mechanism in treatment of rheumatoid arthritis. Jt. Bone Spine..

[9] Juge P.-A., Lee J.S., Lau J., Kawano-Dourado L., Serrano J.R., Sebastiani M., Koduri G., Matteson E., Bonfiglioli K., Sawamura M. (2021). Methotrexate and rheumatoid arthritis associated interstitial lung disease. Eur. Clin. Respir. J..

[10] Lin Y.-J., Anzaghe M., Schülke S. (2020). Update on the pathomechanism, diagnosis, and treatment options for rheumatoid arthritis. Cells.

[11] Feng X., Chen Y. (2018). Drug delivery targets and systems for targeted treatment of rheumatoid arthritis. J. Drug Target..

[12] Zhang A., Lee Y.C. (2018). Mechanisms for joint pain in rheumatoid arthritis (RA): From cytokines to central sensitization. Curr. Osteoporos. Rep..

[13] Wang W., Zhou H., Liu L. (2018). Side effects of methotrexate therapy for rheumatoid arthritis: A systematic review. Eur. J. Med. Chem..

[14] Sebastiani M., Atzeni F., Milazzo L., Quartuccio L., Scirè C., Gaeta G.B., Lapadulag G., Armignaccoh O., Tavioi M., Olivieri I. (2017). Italian consensus Guidelines for the management of hepatitis B virus infections in patients with rheumatoid arthritis. Jt. Bone Spine.

[15] Baniaamam M., Paulus W.J., Blanken A.B., Nurmohamed M.T. (2018). The effect of biological DMARDs on the risk of congestive heart failure in rheumatoid arthritis: A systematic review. Expert Opin. Biol. Ther..

[16] Romero-Sanchez C., Rodriguez C., Santos-Moreno P., Mesa A.M., Lafaurie G.I., Giraldo S.-Q., De-Avila J., Castillo D.M., Duran M., Chalem C.P. (2017). Is the treatment with biological or non-biological DMARDS a modifier of periodontal condition in patients with rheumatoid arthritis?. Curr. Rheumatol. Rev..

[17] Ramiro S., Sepriano A., Chatzidionysiou K., Nam J.L., Smolen J.S., Van Der Heijde D., Dougados M., van Vollenhoven R., Bijlsma J.W., Burmester G.R. (2017). Safety of synthetic and biological DMARDs: A systematic literature review informing the 2016 update of the EULAR recommendations for management of rheumatoid arthritis. Ann. Rheum. Dis..

[18] Uthman I., Almoallim H., Buckley C.D., Masri B., Dahou-Makhloufi C., El Dershaby Y., Sunna N., Raza K., Kumar K., Huijer H.A.-S. (2021). Nurse-led care for the management of rheumatoid arthritis: A review of the global literature and proposed strategies for implementation in Africa and the Middle East. Rheumatol. Int..

[19] Raosoft I. (2020). Sample Size Calculator.

[20] Sepriano A., Kerschbaumer A., Smolen J.S., Van Der Heijde D., Dougados M., Van Vollenhoven R., McInnes I.B., Bijlsma J.W., Burmester G.R., de Wit M. (2020). Safety of synthetic and biological DMARDs: A systematic literature review informing the 2019 update of the EULAR recommendations for the management of rheumatoid arthritis. Ann. Rheum. Dis..

[21] Bottois C., López-Medina C., Dumas S., Julien H., Sephora B., Roux C., Moltó A., Conort O., Dougados M. (2021). POS0273-HPR Pharmacist’s impact on self-management for patients with chronic inflammatory arthritis treated with biological DMARDS. BMJ.

[22] Shakeel S., Hassali M.A., Rehman H., ur Rehman A., Muneswarao J. (2020). Knowledge, attitude, and practice towards biosimilars and interchangeable products: A prescriptive insight by the pharmacists. Int. J. Gen. Med..

[23] Garneau K.L., Iversen M.D., Tsao H., Solomon D.H. (2011). Primary care physicians’ perspectives towards managing rheumatoid arthritis: Room for improvement. Arthritis Res. Ther..

[24] Mok C.C. (2018). Hepatitis B and C infection in patients undergoing biologic and targeted therapies for rheumatic diseases. Best Pract. Res. Clin. Rheumatol..

[25] Wang S., Tseng C., Hsu C., Tung C., Huang K., Lu M., Lai N. (2021). Reactivation of hepatitis B virus infection in patients with rheumatoid arthritis receiving tofacitinib. Int. J. Rheum. Dis..

[26] Littlejohn E.A., Monrad S.U. (2018). Early diagnosis and treatment of rheumatoid arthritis. Prim. Care.

[27] Shirani F. (2019). Primary Care in rheumatoid arthritis. Rheum. Res..

[28] Mathijssen E.G., Vriezekolk J.E., Popa C.D., van den Bemt B.J. (2020). Shared decision making in routine clinical care of patients with rheumatoid arthritis: An assessment of audio-recorded consultations. Ann. Rheum. Dis..

[29] De Mits S., Lenaerts J., Vander Cruyssen B., Mielants H., Westhovens R., Durez P., Elewaut D. (2016). A nationwide survey on patient’s versus physician s evaluation of biological therapy in rheumatoid arthritis in relation to disease activity and route of administration: The be-raise study. PLoS ONE.

[30] Mahlich J., Sruamsiri R. (2017). Preference for shared decision-making in Japanese patients with rheumatoid arthritis. Cogent Med..

[31] Shakeel S., Mohamed A.H., Rehman H., Iffat W., Yasmin R., Farrukh U. (2020). Explanatory Findings of Prescribing Biosimilar Medicines in Oncology Care Settings of Pakistan. Lat. Am. J. Pharm..

[32] Kalkan A., Roback K., Hallert E., Carlsson P. (2014). Factors influencing rheumatologists’ prescription of biological treatment in rheumatoid arthritis: An interview study. Implement. Sci..

[33] Aladul M.I., Fitzpatrick R.W., Chapman S.R. (2018). Healthcare professionals’ perceptions and perspectives on biosimilar medicines and the barriers and facilitators to their prescribing in UK: A qualitative study. BMJ.

[34] Leonard E., Wascovich M., Oskouei S., Gurz P., Carpenter D. (2019). Factors affecting health care provider knowledge and acceptance of biosimilar medicines: A systematic review. J. Manag. Care Spec. Pharm..

[35] Sarnola K., Merikoski M., Jyrkkä J., Hämeen-Anttila K. (2020). Physicians’ perceptions of the uptake of biosimilars: A systematic review. BMJ.

[36] Daei A., Soleymani M.R., Ashrafi-Rizi H., Zargham-Boroujeni A., Kelishadi R. (2020). Clinical information seeking behavior of physicians: A systematic review. Int. J. Med. Inform..

